# The Effect of Gamma-Ray Irradiation on the Physical, Mechanical, and Morphological Characteristics of PVA-Collagen-Chitosan as a Guided Tissue Regeneration (GTR) Membrane Material

**DOI:** 10.1055/s-0042-1753451

**Published:** 2022-10-11

**Authors:** Ira Komara, Agus Susanto, Amaliya Amaliya, Basril Abbas, Yessy Warastuti, Ina Hendiani, Aldilla Miranda, Annisa Pranuditha Erliani

**Affiliations:** 1 Department of Periodontics, Faculty of Dentistry, Universitas Padjadjaran, Bandung, Indonesia; 2 Research Center for Radiation Process Technology, National Research and Innovation Agency (NRIA), Jakarta Indonesia

**Keywords:** gamma irradiation, guided tissue regeneration, polyvinyl alcohol (PVA)

## Abstract

**Objective** The aim of this study was to evaluate the effect of gamma-ray irradiation on the physical, mechanical, and morphological characteristics of the polyvinyl alcohol (PVA)-collagen-chitosan membranes as a guided tissue regeneration membrane material.

**Material and Method** The membrane was fabricated by mixing PVA, collagen, and chitosan using the film casting method. PVA-collagen-chitosan membranes were irradiated with various radiation dose (0, 15, and 25 kGy). Furthermore, it is characterized using Fourier-transform infrared (FTIR) for functional group identification, morphological test was performed using scanning electron microscopy (SEM), and mechanical properties (i.e., tensile strength and elongation) were evaluated using universal testing machine and swelling studies.

**Statistical Analysis** Statistical analysis was performed based on analysis of variance and post hoc with *p* -value < 0.05.

**Result** The FTIR spectrum shows various peaks of functional groups from the PVA-collagen-chitosan membrane. The result of the statistical analysis show changes in tensile strength ( *p* = 0.0004) and membrane elongation ( *p* = 0.000451) at different radiation doses of 0, 15, and 25 kGy. The membrane absorption obtains *p* -value of 0.0193, while the SEM results show that the PVA-collagen-chitosan membrane homogeneously mixed.

**Conclusion** There is an effect of gamma-ray irradiation on tensile strength, elongation, and water absorption of the membranes. Increasing the radiation dose increases the value of tensile strength, while elongation and absorption of the membrane decrease. The PVA-collagen-chitosan membrane has the potential to develop as an alternative membrane for guided tissue regeneration.

## Introduction

Guided tissue regeneration (GTR) is an effective treatment to control disease progression and promote the formation of tooth supporting tissue. 1 GTR has been shown to reduce pocket depth and increase clinical attachment, specifically in infrabony defects. 2 GTR membranes acts as a barrier that can prevent the migration of epithelial cells into the defect area while providing space to guide the regeneration of damaged tissues, such as cementum, periodontal ligament, and alveolar bone. The ideal characteristics of membranes for GTR should be biocompatible, cell exclusion, space maintenance, tissue integration, and ease of use. In the future, membrane is expected to have biological activity to stimulating cell regeneration. 3 Collagen membranes as conventional bioabsorbable membranes are mostly used for GTR materials in clinical practice. 3 However, collagen has poor physical properties. In this study, chitosan and polyvinyl alcohol (PVA) were added, which are expected to improve the physical characteristic of GTR membranes.

Chitosan is a derivative of chitin that has been used in industry, agriculture, food, and medicine. It is hypoallergenic, antimicrobial, biocompatible, and biodegradable. Moreover, chitosan can be processed into nanofibers, gels, membranes, nanoparticles for additional preparations in periodontal treatment, and implants. 4 5 6 7

PVA has also attracted attention for the implementation of other material. It is a water-soluble synthetic polymer with excellent biocompatibility and easy processing for biomedical applications, with tissue-like elasticity and excellent mechanical strength. PVA can improve a material's mechanical properties. 1 8 9 10 ^,^ Different types of PVA can be produced depending on the level of hydrolysis involved, which can affect the physical properties of the polymer and the films made. 10 Mixing collagen with synthetic polymers has the potential to develop into an alternative to GTR membranes.

Gamma-ray irradiation is one of the sterilization methods and has been widely used in the sterilization process of the biomaterial because it can modify material physical properties including GTR membranes. 11 The radiation dose is the most critical parameter to consider during this process, using an inappropriate radiation dose will cause physical and chemical changes of materials. 11 The dose of gamma irradiation required to sterilize medical equipment according to ISO (International Organization for Standardization) 11137 is 15 or 25 kGy. In this study, radiation doses of 0, 15, and 25 kGy were used to determine which dose is the most appropriate for membrane sterilization. 12

This study aims to assess the effect of gamma-ray irradiation on physical, mechanical, and morphological characteristics from the PVA-collagen-chitosan membrane.

## Material and Method

The membrane is made by mixing PVA (5 and 7.5% concentrations), collagen, and chitosan using the film casting method. PVA-collagen-chitosan membranes were irradiated with various radiation doses (0, 15, and 25 kGy). Then, the membrane is tested for various physical properties; tensile strength, elongation, functional groups, morphology, and membrane absorption. The fabrication process and membrane characterization are conducted at Research Center for Radiation Process Technology, National Research and Innovation Agency (NRIA), Jakarta Indonesia.

### Materials

PVA made by Merck, collagen extracted from cow tendons, medical-grade chitosan extracted from shrimp shells, acetic acid, and NaOH solution made by Merck were used. Meanwhile, the equipment used includes gamma-rays (Gammacell 220), Fourier-transform infrared (FTIR) spectrometer (Shimadzu Prestige-21), universal testing machine (UTM), scanning electron microscope (SEM) by Thermoscientific, analytical balance (Aculab BL 210 S Sartorius), magnetic stirrer (Fischer Scientific), glass (measuring cup, Erlenmeyer, and beaker), and polypropylene plastic.

### Preparation of PVA–Collagen–Chitosan Membrane

Preparation of 3% collagen solution in acetic acid, and the overnight homogenization process is performed. Preparation of 2% chitosan solution by mixing 4 g of chitosan, 2 mL acetic acid, and 194 mL aquadest and then the solution is homogenized overnight. PVA solution with 5 and 7.5% concentration are made. Then, the prepared PVA-collagen-chitosan membrane solution is mixed with a ratio of 1:1:1 followed by overnight homogenization. The composite is poured into an acrylic mold of 7.5 × 7.5 cm with a weight of 10 g and dried at room temperature for at least 3 to 4 days to get consistent results. The dry membrane is soaked in NaOH solution for 1 hour and washed until neutral pH. Freezing the membrane in a deep freezer (–80°C) for 24 hours, and then drying the membrane by lyophilization or freeze-drying method with a freeze dryer for 4 hours. The dry membranes are packed in polypropylene plastic bags and irradiated using gamma-rays at radiation doses of 15 and 25 kGy. Two membranes with different composition were prepared: PVA 5%-collagen-chitosan (P1) and PVA 7.5%-collagen-chitosan (P2).

### Functional Group Analysis Using FTIR Spectrophotometer

Irradiated and nonirradiated composite membranes were cut into small pieces (0.2 × 0.2 mm), placed composite membranes on top of potassium bromide (KBr) powder, and then analyzed by FTIR spectrophotometer at wave numbers 4000 to 500 cm ^–1^ . In addition, PVA, collagen, and chitosan powder are also tested using FTIR and analyzed.

### Mechanical Properties Testing of Composite Membrane

The mechanical properties of the composite membrane are tested by measuring the tensile strength and elongation of the membrane. Tests are conducted based on the American Standard Mechanical Testing (ASTM D3039) method using a UTM. The membrane is a standard dumbbell with two clamped ends in an up-down position on the machine. Then, one end of the membrane is pulled up by the machine, and after the middle breaks, the initial distance ( *L*_0_ ) and the break ( *L*_1_ ) are measured.

### Morphological Analysis Using SEM, Element Analysis Using Energy-Dispersive X-Ray

Morphological test was performed using SEM, and the presence of PVA-collagen-chitosan element was performed using energy-dispersive X-ray (EDX).

### Membrane Water Absorption Test

The irradiated and nonirradiated composite membranes were cut into 20 × 20 mm. Then, the membrane is dried in an oven at 40°C for 24 hours to a constant weight ( *W*_0_ ). Moreover, the composite membranes are immersed in distilled water with a volume of 50 mL and soaked with variations in immersion time between 30 seconds to 24 hours to confirm the perfect swelling. Then, the sample is placed on filter paper to remove water on the membrane surface and then weighed ( *W*_1_ ). Water absorption is calculated using the equation:



where *W*_0_ is dry composite weight (g) and *W*_1_ is the weight of the sample after being soaked (wet composite weight). 13

### Membrane Color Test

The membrane surface color test is performed using a Minolta CR-200 chroma meter, and the measurement begins by selecting the menu on the chroma meter tool to use the *L* , *a* , and *b* measurement scale. Furthermore, the membrane sample is placed on white paper, and the optical head is placed vertically above the sample before pressing the start button. The data from the sample color measurements can be observed in the processor section in Commission Internationale de l'Eclairage (CIE) L* a* b* color units. Subsequently, measurements are performed three times to calculate the average. Color testing is performed before storage and after storing samples at 4°C for 1.5 months.

### Statistical Analysis

Analysis of the effect of gamma rays on PVA-collagen-chitosan membranes is performed using analysis of variance (ANOVA) and post hoc with *p* -value < 0.05 on quantitative data, including tensile strength, elongation, and water absorption of the membrane.

## Results

**Fig. 1:**
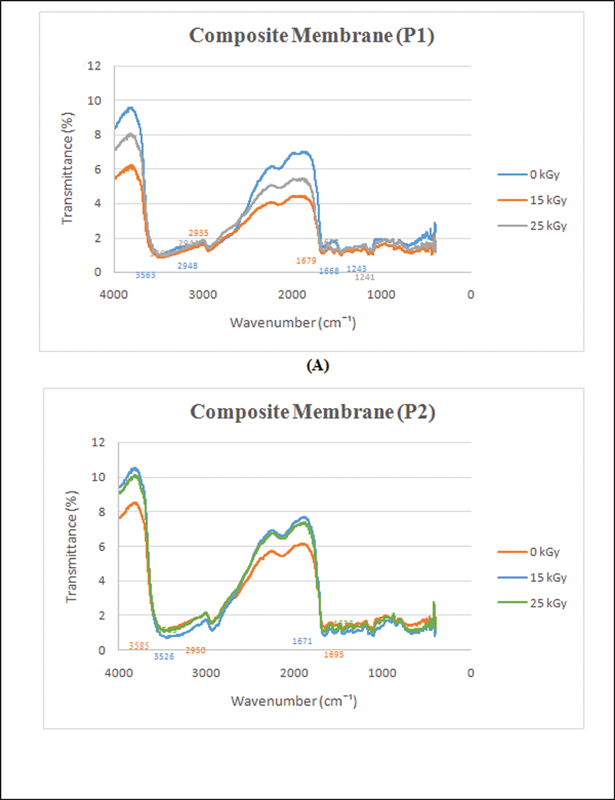
Fourier-transform infrared (FTIR) result of P1 composite membrane ( **A** ), and P2 composite membrane ( **B** ).

The FTIR measurement examines the changes in the chemical structure of the PVA-collagen-chitosan and the effect of gamma-ray irradiation on the functional groups, and the spectrum irradiated with doses of 15 and 25 kGy is presented in Fig. 1 . With the increasing radiation dose, there is no change in the peaks of functional groups due to gamma irradiation compared with membranes treated with not irradiated (0 kGy). The characteristics of the FTIR spectrum of the 5 and 7.5% PVA-collagen-chitosan membrane functional groups are presented in Tables 1 and 2 .

**Table 1:** FTIR result of composite membrane (P1)

Functional group	Wavenumber (cm ^–1^ )	Wave number (cm ^–1^ )		
		0 kGy	15 kGy	25 kGy
OH, NH	3750–3000	3563	3563	3562
CH3	3300–2900	2948	2935	2944
C = O, NH2	1820–1660	1668	1679	1673

Abbreviation: FTIR, Fourier-transform infrared.

**Table 2:** FTIR result of composite membrane (P2)

Functional group	Wavenumber (cm ^–1^ )	Wave number		
		0 kGy	15 kGy	25 kGy
OH, NH	3750–3000	3585	3526	3563
CH3	3300–2900	2950	2950	2950
C = O, NH2	1820–1660	1659	1671	1636

Abbreviation: FTIR, Fourier-transform infrared.

The wavenumber characteristics of collagen are found in three absorption bands, wavenumbers 1643, 1543, and 1240 cm ^–1^ , representing the amide bands 1, 2, and 3, respectively. However, the amide 3 absorption band (1240 cm ^–1^ ) is the main characteristic of the collagen amide band. Meanwhile, PVA characteristics obtained at 3274 and 1674 cm ^N1^ are –OH stretching and bending vibrations. The band at the peak of the wavenumber 2942 cm ^–1^ is an asymmetric stretching vibration of the methylene group (–CH2). The stretching vibration of H-NH at 3286 to 3104 cm ^–1^ , NH2 at 1645 cm ^–1^ , and C-H bending vibration of CH2 at 1423 cm ^–1^ are all part of the usual chitosan spectrum.

**Fig. 2:**
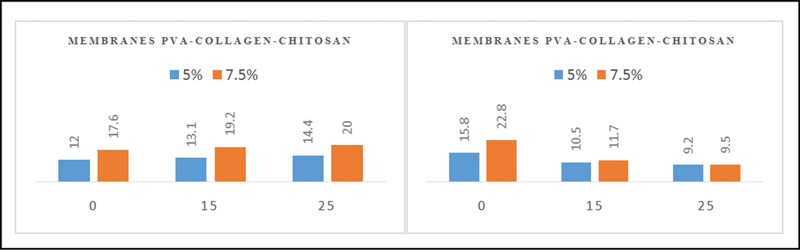
The tensile strength value of the polyvinyl alcohol (PVA)-collagen-chitosan membrane 5% and 7.5% ( **A** ), the value of the elongation of the PVA-collagen-chitosan membrane 5% and 7.5% ( **B** ).

Tensile strength and elongation at break are the maximum stresses that a material can resist when stretched before it breaks. 13 The tensile strength and elongation results are presented in Fig. 2 .

Fig. 2 shows that when the radiation dose increases, the tensile strength value increases, and the elongation value decreases. PVA-collagen-chitosan 5% (P1) – 0 kGy, membrane gives the smallest value with an average of 12 Mpa compared with other treatments. On the other hand, the most significant value is in the PVA-collagen-chitosan membrane 7.5% (P2) – 25 kGy with an average of 24 Mpa. For elongation result, the smallest value is in PVA-collagen-chitosan membranes 5% (P1) - 25 kGy and PVA-collagen-chitosan membranes 7.5% (P2) - 25 kGy.

**Fig. 3:**
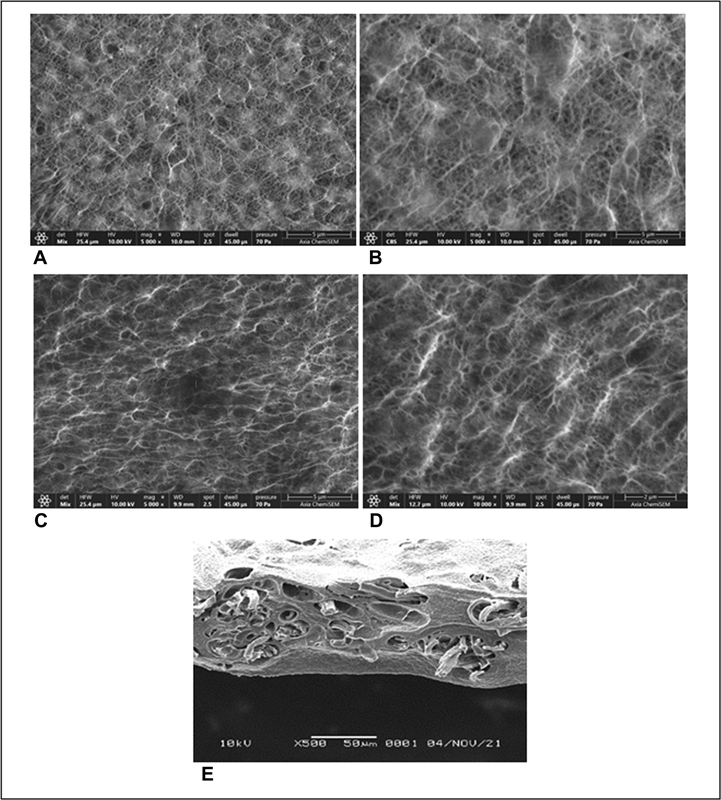
Scanning electron microscopy (SEM) results of composite membrane 5%-0 kGy with magnification 5,000× ( **A** ), composite membrane 5%-0 kGy with magnification 10,000× ( **B** ), composite membrane 7.5%-0 kGy with magnification 5,000× ( **C** ), composite membrane 7.5%-0 kGy with magnification 10,000× ( **D** ), composite membrane 7.5%-25 kGy cross-sectional ( **E** ).

**Fig. 4:**
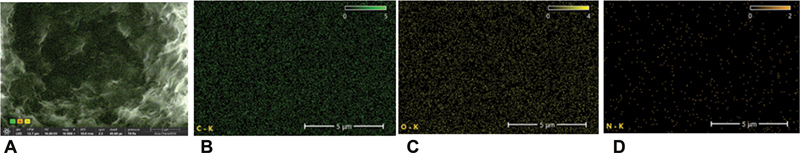
Energy-dispersive X-ray (EDX) result of membrane polyvinyl alcohol (PVA)-collagen-chitosan. ( **A** ) Distribution of carbon element, ( **B** ) distribution of oxygen element, and ( **C** ) distribution of nitrogen element.

SEM is an electron microscope designed to observe the morphology of solid objects surfaces directly. EDX is a technique of elemental analysis associated to electron microscope that reveals the presence of nanoparticle. SEM and EDX results are presented in Figs. 3 and 4 .

**Fig. 5:**
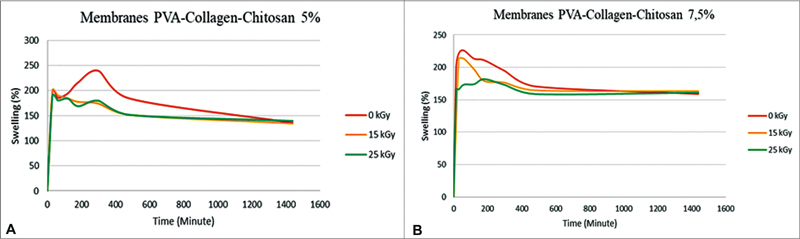
Water absorption of polyvinyl alcohol (PVA)-collagen-chitosan membrane 5% ( **A** ), water absorption of PVA-collagen-chitosan membrane 7.5% ( **B** ).

The ability of the membrane to absorb water is one of the important parameters. 14 The measurement results of water absorption on the PVA-collagen-chitosan membrane irradiated at a dose of 0, 15, and 25 kGy are shown in Fig. 5 . Based on the results of the ANOVA analysis ( *p* < 0.05), the significance value is smaller than 0.05. Therefore, it is an effect of radiation dose on membrane absorption. Then a follow-up post hoc test was used to determine the significant difference in the variation of radiation dose.

**Fig. 6:**
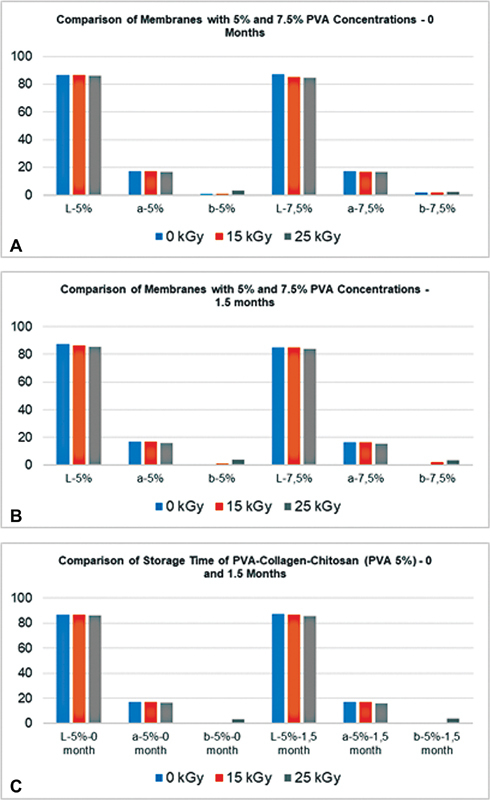
The color graph of P1 and P2 composite membranes at 0 months storage ( **A** ), the comparison of P1 and P2 composite membranes after 1.5 months storage ( **B** ), the comparison storage time (0 and 1.5 months) of P1 ( **C** ).

The color on the surface of the PVA-collagen-chitosan membrane is observed with a chroma meter. The tests were conducted to compare the color of the 5 and 7.5% PVA-collagen-chitosan composite samples after being irradiated (15 and 25 kGy). The measurements are performed at 0 and 1.5 months of storage, as presented in Fig. 6 .

**Fig. 7:**
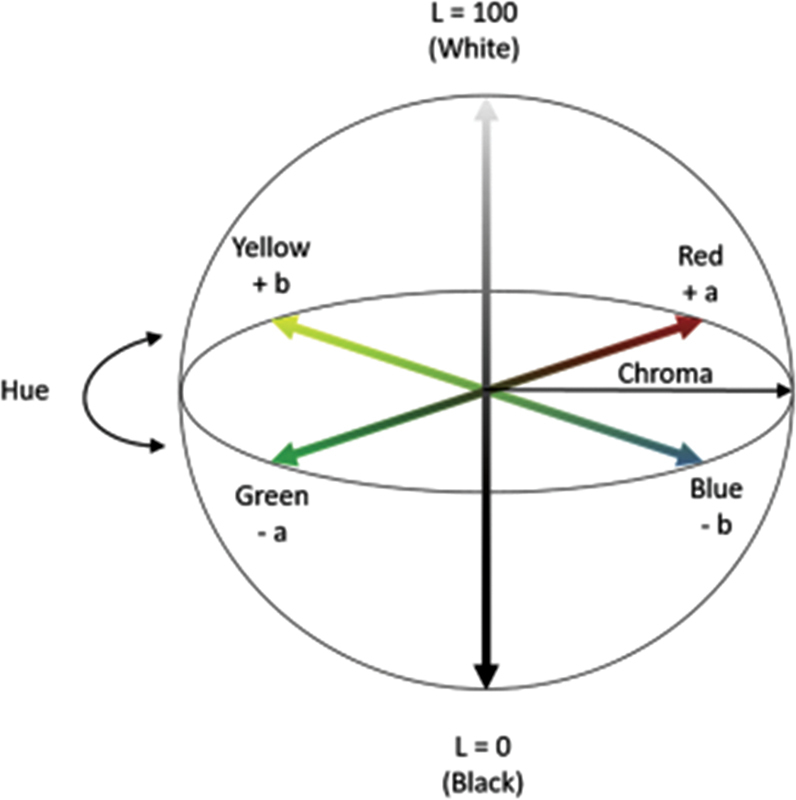
Commission Internationale de l'Eclairage (CIE) LAB color quantity determination chart. 23

The graphs in Fig. 6 show the results of the color change test on the PVA-collagen-chitosan membrane. The *L* value indicates the brightness of the color, while the *a* and *b* values indicate the chromaticity coordinates on the color space diagram ( Fig. 7 ). The following are the findings of the color change test: The surface color of the sample altered slightly when the irradiation dosage was increased in both the 5 and 7.5% PVA composite membranes.

The composite membranes results are white and the closer the *L* value is to 100, the lighter/whiter the sample will be. For example, at 0 months storage, the composite membrane with 0 kGy dose irradiation show a slightly higher *L* value when compared with 15 and 25 kGy doses (86.8 for 5% PVA and 87.1 for 7.5% PVA 0 kGy dose and at doses of 25 kGy are 86.3 and 84.7 for 5 and 7.5% PVA, respectively). Meanwhile, as the value of *b* increases ( *b* + ), the color yellow appears in both concentrations of *b* value of 3.3 for 5% PVA irradiated at a dose of 25 kGy compared with a nonirradiated dose of 0 kGy ( *b* = 0.8) as well as the PVA concentration of 7.5% is *b* = 2.3 (25 kGy) compared with *b* = 1.7 (0 kGy). The same results are also shown in the color test on samples after 1.5 months of storage. The radiation dose of 25 kGy changed the color of the sample to a slightly more yellowish color; however, the change is not very significant when viewed with the eye, as seen in Fig. 6C . The *L* and *b* remained relatively unchanged after the membranes were stored for 1.5 months, showing no color change.

## Discussion

Chitosan is a popular material for GTR because it is biocompatible, biodegradable, antimicrobial, has similar structure to bone extracellular matrix, and cost-effective. PVA is a polymer with good chemical and mechanical properties and has either been used alone or in combination with biodegradable polymer to form scaffolds for various application. In this study, chitosan and PVA were added with collagen which are expected to improve the physical characteristic of GTR membranes. 14 15

Gamma-rays irradiation was used to sterilize the membrane to produce sterile and ready to use membrane. The method commonly used for sterilization is gamma rays from cobalt-60 because it is practical (easy to use), predictable, has good enough penetrating ability, and the sample can be sterilized after final packaging. 16 17 Furthermore, the membranes are characterized using FTIR for functional group identification, morphology test by SEM, mechanical properties test with UTM, and water absorption test.

The FTIR results are presented in Fig. 1 . In this study, two radiation doses (15 and 25 kGy) were used to determine which dose was the most appropriate for membrane sterilization. From FTIR results, it was found that there was no change in the absorption peak at the two doses (15 and 25 kGy). This means that the two radiation doses do not change the functional groups of the membrane.

Tensile strength and elongation at break indicate the membranes' strength and elasticity, which are important physical parameters in supporting their application. 13 Membranes are generally used in areas of the tooth that require frequent movements, such as chewing and biting. Therefore, the membrane should be elastic, flexible, and strong enough so that it is resistant to tension and pressure when applied to the tooth area. The radiation used can cause two interaction mechanisms in the polymer: chain-breaking, which decreases the value of maximum tensile strength and elongation, and cross-linking, which increases maximum tensile strength but decreases the elongation value until breaking. 17 18 The most dominant response in this study is cross-linking because the tensile strength value increased as the radiation dose increased. According to the data, the elongation value decreased on both composite membranes.

Based on SEM analysis, it is found that PVA, collagen, and chitosan are homogeneously mixed ( Fig. 3 ). The membrane exhibits pore microarchitecture and interconnectivity over the entire surface that provides space for vascularization. The membrane's outer surface is a physical barrier for the migration of epithelial or connective tissue cells. However, it remains permeable to the passage of macromolecules needed to provide nutrients used for tissue repair. 19 20 Based on cross-sectional SEM result of composite membrane, it showed the interconnected porous that formed from a mixture of PVA/chitosan and the inner surface of the membrane showed some possible collagen fiber ( Fig. 3E ).

The EDX microanalysis is a technique of elemental analysis associated to electron microscope to show the presence of elements in the sample. As per EDX results, we obtained the distribution of carbon (C), nitrogen (N), and oxygen (O) on both composite membranes as presented in Tables 3 and 4 . The carbon that was found in the EDX test is considered organic component. 21

**Table 3:** EDX result of composite membrane (P1)

Element	Atomic %	Atomic % error	Weight %	Weight % error
C	46.8	0.2	40.1	0.2
N	4.8	0.5	4.8	0.5
O	48.3	0.4	55.1	0.5

Abbreviation: EDX, energy-dispersive X-ray.

**Table 4:** EDX result of composite membrane (P2)

Element	Atomic %	Atomic % error	Weight %	Weight % error
C	46.3	0.5	39.5	0.4
N	3.6	1.3	3.6	1.3
O	50.1	1	56.9	1.1

Abbreviation: EDX, energy-dispersive X-ray.

The ability of the membrane to absorb water is presented in Fig. 5 . Water absorption is one of the essential parameters for GTR membranes. The smallest water absorption occurs in the irradiated membrane using a dose of 25 kGy. The decrease in water absorption on the PVA-collagen-chitosan membrane irradiated with the highest dose (25 kGy) can be caused by the degradation of chitosan. From the two variations of PVA composition, it can be seen that the higher percentage of PVA indicates lower water absorption, this condition is related to density. The high density caused by the PVA molecules filling the pores, there is a lot of possibility in water blockage and reducing water absorption. 22

The last characterization tested the color change of the irradiated and nonirradiated membranes after 1.5 months of storage. The colorimeter measures the quantification of colors that reflect human vision created under the standards of the CIE, which is the worldwide authority on light and color. 23 Moreover, color quantification is represented by the XYZ color system in the form of monochromatic standards of red, green, and blue indicated by CIELAB or CIE L* a* b*.

Fig. 6 shows the CIELAB color quantity determination diagram, and the system represents the quantitative relationship of color on three axes. The *L* value represents brightness on the vertical axis from 0 (black) to 100 (white). Furthermore, *a* and *b* values indicate the chromaticity coordinates on the color space diagram. For example, the value indicates the red-green component, where a positive or negative *a* indicates red or green. Meanwhile, the yellow-blue component is indicated by the value of *b* , where a positive or negative *b* indicates a yellow and blue color.

## Conclusion

PVA-collagen-chitosan membrane has the potential to develop as an alternative membrane for GTR. There is an effect of gamma-ray irradiation on tensile strength, elongation, and water absorption of the membranes. Increasing the radiation dose increases the value of tensile strength, while elongation and absorption of the membrane decrease. From FTIR results, it was found that there was no change in the absorption peak at the two doses (15 and 25 kGy), this means that two radiation doses do not change the functional groups of the membrane. Meanwhile, based on the SEM test it is found that PVA, collagen, and chitosan are homogeneously mixed. The membrane exhibits the pore microarchitecture and some possible interconnectivity that provides space for vascularization.
